# Analysis of clinical pharmacists’ interventions in a rehabilitation setting

**DOI:** 10.1080/20523211.2025.2450593

**Published:** 2025-04-25

**Authors:** Lina Naseralallah, Zahra Noureddine, Afif Ahmed, Moza Al Hail, Somaya Koraysh

**Affiliations:** Department of Pharmacy, Hamad Medical Corporation, Doha, Qatar

**Keywords:** Drug-related problems, clinical pharmacist, pharmacist interventions, rehabilitation, medication management

## Abstract

**Background:**

To elucidate the role of clinical pharmacists in rehabilitation programmes by examining the type, severity, medications involved, and the level of acceptance of pharmacists’ interventions

**Methods:**

A cross-sectional study was conducted at the Qatar Rehabilitation Institute (QRI) in Doha, Qatar. Clinical pharmacists’ interventions and their underlying rationales were categorised by the type of intervention using a validated classification system. The severity of these interventions was assessed using the National Patient Safety Agency (NPSA) Risk Matrix. Linear regression and chi-square analyses were employed to examine the relationships between patient-related and medication-related characteristics and the pharmacist interventions.

**Results:**

A total of 3,807 clinical pharmacists’ interventions involving 815 patients were collected and analysed. The majority of patients (*n* = 501, 61.5%) had three or more interventions. Findings indicated that most interventions were addressing pharmacological strategy (*n* = 1670, 43.9%) and drug quantity (*n* = 1166, 30.7%). The most frequently reported intervention subtypes included dose optimisation (*n* = 749, 19.7%), additional drug therapy (*n* = 673, 17.7%), and medication discontinuation (*n* = 476, 12.5%). Cardiovascular agents were involved in 37.1% of the interventions, followed by endocrine medications (17.1%) and centrally acting agents (11.7%). A significant proportion of interventions were deemed to have moderate severity (79.8%). Statistical analysis revealed a positive linear correlation between age and the number of interventions per patient (*p* < 0.001); with no significant difference in the severity of interventions between adult and elderly patients (*p* = 0.09).

**Conclusion:**

This study highlights the diverse roles of clinical pharmacists in the rehabilitation field. The unique complexity of rehabilitation patients creates a challenging environment for clinical pharmacists, requiring adherence to fundamental practice principles while customising approaches to address individual patient needs. Further research is needed to assess the impact of these interventions on clinically significant outcomes.

## 1. Background

The Pharmaceutical Care Network Europe (PCNE) defines a drug-related problem (DRP) as an event or circumstance involving drug therapy that interferes with desired health outcomes (*Pharmaceutical Care Network Europe classification for drug-related problems*, 2020). DRPs are associated with preventable patient harm and have profound cost implications for healthcare systems and the economy (Leendertse et al., 2011; Rajakumar et al., 2024). Globally, studies have reported that the incidence of DRPs among hospitalised patients has ranged from 15.5% to 81.0% (Blix et al., 2004; Garin et al., 2021; Peterson & Gustafsson, 2017). Hospitalised patients are specifically vulnerable to DRPs due to their acute medical condition, presence of co-morbidities, polypharmacy and frequent changes to their medication regimens (Blix et al., 2004). Fortunately, most DRPs can be avoided with appropriate measures to identify and resolve them (Howard et al., 2007; Leendertse et al., 2008).

Clinical pharmacists working in a hospital setting are ideally positioned to prevent DRPs. A pharmacist role ranges from reviewing medication orders, advising on drug therapy, monitoring patient response, educating healthcare professionals and patients, collaborating on care plans and conducting medication reconciliations (Rahayu et al., 2021). Research has shown that integrating clinical pharmacists into multidisciplinary teams improves the detection of clinically significant DRPs, hence enhances patient outcomes and reduces medication errors (Abunahlah et al., 2018; Delgado Silveira et al., 2015; Guignard et al., 2015; Studer et al., 2023; Viktil & Blix, 2008). Multiple studies have evaluated the clinical and economic impact of pharmacists’ intervention in various hospital settings, including hematology, surgical, medical, acute care and intensive care units (Chen et al., 2020; Cvikl & Sinkovič, 2020; Elnour et al., 2022; MacTavish et al., 2019; Naseralallah et al., 2023; Naseralallah et al., 2024); however, their impact in a rehabilitation settings remains unexplored.

Over the past years, we have witnessed the emergence of clinical pharmacy services in rehabilitation settings (Willoch et al., 2012; Yoshimura et al., 2022). The unique complexity of rehabilitative patients poses a challenge on the multidisciplinary team in terms of medication management. Patients undergoing rehabilitation often experience numerous changes in medication regimens throughout their prolonged hospital stay exposing them to an increased risk of DRPs and medication errors (Yoshimura et al., 2022). Utilising clinical pharmacists in this setting is of utmost importance to ensure optimal patient care and appropriate prescribing.

To further stress the importance of incorporating a clinical pharmacist in the rehabilitation team, it is worth noting that rehabilitation doctors, also known as physiatrists, often have a background in physical medicine and rehabilitation (PM&R). Their training typically emphasises musculoskeletal, neurological, and rehabilitative care making them well suited to manage functional impairment and disabilities rather than complex medical conditions (*Overview of Physical Medicine and Rehabilitation (PM&R)*, 2024). This highlights the significance of working closely with clinical pharmacists, who are the medication experts, to ensure thorough and safe patient care.

In 2016, Qatar launched the region’s largest tertiary rehabilitation hospital; the Qatar Rehabilitation Institute (QRI). QRI is a specialised inpatient rehabilitation facility focused on providing rehabilitation services for patients with neurological and musculoskeletal conditions. The institute offers comprehensive programmes designed to support recovery and improve patients’ quality of life. The length of stay at QRI is typically long, as patients require intensive physical and mental rehabilitation to reintegrate into the community. QRI has approximately 193 beds and offers five main rehabilitation programmes – Stroke, Traumatic Brain Injury, Spinal Cord Injury, Pain Management and Pediatric Rehabilitation. These programmes are delivered by a multi-disciplinary team of clinicians, including a clinical pharmacist (*Qatar Rehabilitation Institute*, 2024). The role of clinical pharmacy in rehabilitation has not yet been fully defined in the literature. However, a recent review stressed the importance of pharmacist-led medication management in maximising patients’ rehabilitation outcomes (Yoshimura et al., 2022). Although some studies have explored the role and impact of pharmacists in other rehabilitation settings, such as cardiac or post-intensive care programmes, or have included pharmacist-led outpatient clinics (Casper et al., 2019; MacTavish et al., 2019; Nathans et al., 2020), to date, no studies have specifically analysed and characterised clinical pharmacists’ interventions in an inpatient neurological and musculoskeletal rehabilitation setting. This study aims to elucidate the role of clinical pharmacists in rehabilitation programmes by examining the type, severity, medications involved, and the level of acceptance of pharmacists’ interventions. The findings from this study will provide an insight into the extent of clinical pharmacists’ involvement in the rehabilitation team and will inform potential areas for improvement.

## 2. Methods

### 2.1. Ethics approval

Ethics approval for the study was obtained from the Medical Research Center (MRC) at Hamad Medical Corporation (HMC) in 2024 (MRC-01-24-111).

### 2.2. Study design and setting

This cross-sectional study was conducted at QRI in Doha, Qatar. QRI delivers top-tier, integrated rehabilitation services for patients with a range of neurological and physical disabilities. The facility generally has longer patient stays than other hospitals within HMC, as patients need intensive physical and mental rehabilitation to effectively reintegrate into the community. This extended care offers ample opportunities to improve a variety of health outcomes for admitted patients. Given this, the Pharmacy Department, in partnership with QRI administration, introduced a clinical pharmacy service. This service is provided by three clinical pharmacists who offer ward coverage from Sunday to Thursday. These pharmacists oversee patient care from admission through discharge, collaborating with the healthcare team on various tasks, such as attending rounds, optimising medications, conducting medication reviews, performing reconciliation, and addressing drug information queries.

### 2.3. Data processing

Clinical pharmacists record their interventions electronically via an ad-hoc note within the electronic medical records (EMR) system. An information technology pharmacist retrieved the interventions conducted in all of QRI units over a one-year period (January 2023 to January 2024). The collected data was anonymised and processed using Microsoft Excel. Data cleansing was applied to remove duplicate entries, incomplete records, or unclear documentation. Categorisation was initially performed by one author (LN), with independent verification by a second author (SK). The two researchers who reviewed and categorised the interventions were not members of the QRI clinical pharmacy team. Furthermore, any identifying information (e.g. name or corporation number) of the performing pharmacist was blinded prior to the review and categorisation process, ensuring that the researcher responsible for this task was unaware of which pharmacist had performed the interventions.

Pharmacist interventions and their underlying rationale were quantified and categorised based on the type of intervention. The classification system, adapted from prior studies with modifications to incorporate frequently occurring interventions (Faus et al., 2007; Haque et al., 2021), divided the interventions into five main groups: (1) pharmacological strategy (i.e. any drug change, including replacement, addition, or discontinuation), (2) drug quantity (adjustments to dose, frequency, duration, or administration schedule), (3) monitoring (i.e. the ongoing assessment of laboratory parameters, diagnostic test, etc.), (4) documentation (i.e. to updating key information to assess the appropriateness of a medication or dosing regimen), and (5) drug information (i.e. responding to drug-related inquiries from other healthcare providers). Two additional categories: ‘patient education’ and ‘medication therapy management’ were also added. Each group was further subdivided into categories (e.g. optimal dose or frequency under drug quantity).

The medications involved in the interventions were categorised into 16 classes: anti-infective agents, cardiovascular drugs, endocrine system and hormonal agents, gastrointestinal drugs, analgesics/anti-inflammatory drugs, anti-neoplastic and immunosuppression, blood derivatives and immunoglobulins, central nervous system agents, fluids and electrolytes, vitamins and nutritional agents, eye, ear, nose and throat (EENT) drugs, musculoskeletal and joint disease drugs, respiratory tract agents, urinary-tract disorders agents, total parenteral nutrition (TPN), and vaccines. Each medication was recorded exactly as reported by the pharmacists when documenting their interventions; thus, these medication categories were derived from the classification system outlined in the clinical pharmacist intervention note within the EMR system.

The severity of the interventions was assessed using a 1–5 scale based on the potential consequences of the associated risk. This scoring system was adapted from the National Patient Safety Agency (NPSA) Risk Matrix (*A risk matrix for risk managers*, 2008). A score of 1 represents an intervention with no or minimal clinical impact, while a score of 5 indicates an intervention that could potentially prevent an organ- or life-threatening event (Table 1) (MacTavish et al., 2019).

Clinical pharmacists also document the outcome of their interventions, noting whether they were accepted or rejected by the surgeon.

**Table 1.** Intervention severity scale.

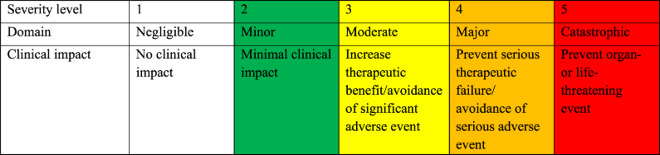

### 2.4. Data analysis

The collected data were analysed with SPSS software (version 22.0) and reported as frequencies, means with standard deviations (SD), or medians with interquartile ranges (IQR), depending on the results of normality testing.

Descriptive statistics were employed to quantify and characterise the pharmacists’ interventions. The chi-square test was used to examine the relationship between age categories (below 65 years old vs. 65 years old and above) and the severity of the interventions. Additionally, linear regression was conducted to explore the association between age and gender with the number of interventions per patient. A 2-sided *P*-value of <0.05 was considered statistically significant.

## 3. Results

### 3.1. Characteristics of clinical pharmacists’ interventions

Characteristics of included patients and interventions are illustrated in Table 2. In this study, 815 patients were included, 76% of which were males. Around 38% of patients were admitted for post-stroke rehabilitation programme (*n* = 310, 38%). The median age of included patients was 49 (IQR 21) years. Elderly patients (65 years or above) constituted 19.6% (*n* = 160) of the patients included. In 2023, a total of 3,939 pharmacist interventions were identified, of which 132 cases were excluded from the study due to unclear or incomplete information. This left a final sample of 3,807 interventions included in the analysis.

**Table 2.** Demographics from included interventions.

Age (median, IQR)*		49	21
Elderly (65 years or above) (*n*, %)		160	19.6%
Gender (*n*, %)	Male	619	75.95%
	Female	196	24.05%
Number of patients (*n*)		815	
Admission unit (*n*, %)			
Stroke rehabilitation unit		310	38.0%
Female rehabilitation unit		188	23.1%
Slow stream for neurological disorders unit		117	14.4%
Traumatic brain injuries unit		110	13.5%
Spinal cord injury unit		90	11.0%
Number of interventions		3807	
Number of interventions per patient (median, IQR)*		3	4
1–2 interventions (*n*, %)		314	38.5%
3 or more interventions (*n*, %)		501	61.5%
Prescriber's response (*n*, %)	Accepted	3651	95.90%
	Rejected	156	4.10%

*Not normally distributed *p* < 0.05.

A median of 3 interventions (IQR 4) were recorded per patient. Specifically, 38.5% (*n* = 314) of patients had 1 or 2 pharmacy interventions affecting their therapeutic plans, while 61.5% (*n* = 501) had 3 or more interventions. There was a significant positive correlation between patients’ age and the number of interventions; for each additional year of age, the number of interventions per patient increased by 0.046 (95% CI 0.027–0.065, *p* < 0.001). Gender, however, did not correlate with the number of interventions per patient (*p* = 0.294).

### 3.2. Classification of interventions

An overview of identified interventions is described in Table 3. The types and subtypes of interventions, along with the reasons for them and clinical examples, are described. Majority of the interventions (*n* = 1670, 43.9%) were addressing pharmacological strategy, followed by drug quantity (*n* = 1166, 30.7%), monitoring of laboratory values and diagnostics (*n* = 482, 12.7%), medication therapy management (290, 7.6%), drug information inquiries (*n* = 188, 4.9%), incomplete weight documentation (*n* = 6, 0.2%), and patient education about adherence (*n* = 5, 0.1%).

**Table 3.** Overview of interventions in QRI during 2023.

Intervention category	Subcategory	*n*	%	Reason for intervention	*n*	%	Examples
Pharmacological strategy (*n* = 1670, 43.9%)	Additional Therapy Required	673	17.7%	Untreated condition	207	30.7%	Anemia, *n* = 165(80%); Diabetes, *n* = 17(8%); Culture, *n* = 8(3%); Hypertension, *n* = 6(3%); Dyslipidemia, *n* = 5(2%); Gout, *n* = 2(1%); Hyperthyroidism, *n* = 1(1%); Osteoporosis, *n* = 1(1%); Prediabetes, *n* = 1(1%); CVA, *n* = 1(1%)
				Uncontrolled condition	154	22.8%	Diabetes, *n* = 70(45%); Hypertension, *n* = 68(44%); Heart failure, *n* = 8(5%); other, *n* = 5(3%); Ischemic heart disease, *n* = 3(2%)
				Sequelae of disease	113	16.8%	Spasticity/pain, *n* = 26(23%); Depression, *n* = 25(22%); Aphasia, *n* = 24(21%); Cognitive delay, *n* = 20(18%); Dizziness, *n* = 15(13%); Anxiety, *n* = 2(2%); Tremor, *n* = 1(1%)
				Inpatient complication	100	14.8%	Electrolyte imbalance, *n* = 31(31%); Urinary tract infection, *n* = 22(22%); Tachycardia, *n* = 10(10%); Allergy, *n* = 7(7%); others, *n* = 13(12%); Acute kidney injury, *n* = 5(5%); Infection signs, *n* = 5(5%); GI symptoms, *n* = 4(4%); Edema, *n* = 3(3%)
				Prevention/prophylaxis	87	12.9%	Venous thromboembolism prophylaxis, proton pump inhibitor for patients on DAPT
				Optimise management	12	1.8%	Adding spironolactone for newly diagnosed resistant hypertension
	Discontinue medication	476	12.5%	No longer needed	211	44.4%	Discontinuing ondansetron as patient has no active vomiting episodes
				Not indicated	124	26.1%	Discontinue prednisolone prior to imaging with contrast as no history of allergy reported
				Duplicate therapy	85	17.9%	Duplicate PPI coverage
				Adverse Drug Event	21	4.4%	Discontinue lisinopril due to induced hyperkalemia
				Drug-Disease Interaction	17	3.6%	Discontinue pioglitazone in diabetic patient with history of heart failure
				Drug-Drug Interaction	13	2.7%	Discontinue metoclopramide as it can increase CNS depressive symptoms of levetiracetam
				Other	5	1.1%	Inpatient complication of hypotension
	Hold/Resume	244	6.4%	Omitted home medications	94	39.3%	Resume diabetic home medications
				Kidney function	31	13.0%	Hold valsartan in view of Acute kidney injury
				Liver function	30	12.6%	Hold atorvastatin due to 5x increase in liver enzymes
				Adverse Drug Event	22	9.2%	Hold enoxaparin due to GI bleeding
				Perioperative care	21	8.8%	Hold apixaban 48 hours prior to craniotomy procedure, resume after 24 hours if clinically stable
				Renew order	19	7.9%	Renewal of VTE prophylaxis agent
				Inpatient complication	25	10.5%	Hypotension, *n* = 15(60%); other, *n* = 7(28%); Hypoglycemia, *n* = 2(8%); Electrolyte imbalance, *n* = 1(4%)
				Uncontrolled condition	2	0.8%	Hypertension, *n* = 1
	Alternative Therapy	230	6.0%	Optimise management	82	34.9%	Switch amlodipine to lisinopril in a patient with coronary artery disease
				Availability of product	69	29.4%	Switch Olmesartan to equivalent valsartan dose as the former is not in HMC formulary
				Kidney function	20	8.5%	Switch enoxaparin to heparin as CrCl <30 ml/min
				Adverse Drug Event	14	6.0%	Switch mirtazapine to alternative antidepressant in a morbidly obese patient
				Drug-Drug Interaction	14	6.0%	Switch atorvastatin to rosuvastatin due to interaction with rifampicin
				Culture	10	4.3%	Switch nitrofurantoin to ciprofloxacin due to Pseudomonas in urine culture
				Liver function	5	2.1%	Switch paracetamol to PRN NSAID in view of liver injury
				Infection signs	4	1.7%	Escalate ertapenem to meropenem in view of worsening symptoms
				Drug-Disease Interaction	3	1.3%	Switch metoclopramide to ondansetron as the former can reduce seizure threshold
				NGT inserted	3	1.3%	Switch bisacodyl from Oral to rectal in view of NGT insertion
				Uncontrolled condition	3	1.3%	Diabetes, *n* = 2; Hypertension, *n* = 1
				Pregnant	2	0.9%	Switch heparin infusion to enoxaparin in pregnant lady with PE
				Other	1	0.4%	Allergy, *n* = 1; patient convenience, *n* = 1
	Formulation Selection	47	1.2%	NGT inserted	14	29.8%	To change all active oral medications to NGT compatible forms
				No sufficient response	9	19.1%	not responding to oral form of iron, to switch to IV form
				Availability of product	6	12.8%	Diphenhydramine PO to IM
				Not on NG tube	6	12.8%	To change all active NGT medications to regular PO forms
				Difficulty swallowing	4	8.5%	To change enteric coated form of aspirin to dispersible tablet form in view of dysphagia
				Interchanging oral dosage forms	4	8.5%	To change metformin to extended release form as per patient request
				Oralise	4	8.5%	To change IV order of ondansetron to oral form as patient is tolerating oral administration
Quantity of drug (*n* = 1166, 30.7%)	Optimum Dose	749	19.7%	Uncontrolled condition	298	39.7%	Hypertension, *n* = 175(58%); Diabetes, *n* = 120(41%); Hypothyroidism, *n* = 1(1%); Ischemic heart disease, *n* = 1(1%); other, *n* = 1(1%)
				Inpatient complication	161	21.5%	Hypotension, *n* = 99(61%);Hypoglycemia, *n* = 40(25%); Tachycardia, *n* = 13(7%); Bradycardia, *n* = 3(2%); Allergy, *n* = 1(1%); Edema, *n* = 1(1%); Electrolytes imbalance, *n* = 1(1%); Urinary tract infection, *n* = 1(1%); other, *n* = 2(1%)
				Optimise management	116	15.5%	Optimise warfarin dose based on INR readings
				Sequelae of disease	77	10.3%	Depression, *n* = 28(36%); Spasticity/pain, *n* = 28(36%); Aphasia, *n* = 9(12%); Cognitive delay, *n* = 6(8%); Dizziness, *n* = 5(6%); Anxiety, *n* = 1(1%)
				Weight	37	4.9%	Adjust heparin prophylactic dose in morbidly obese patient
				Exceeded maximum dose	16	2.1%	Not to exceed maximum dose of 400 mg/day for spironolactone in patient with primary aldosteronism
				Kidney function	14	1.9%	Adjust amantadine to 100 mg daily in view of reduced kidney function
				Liver function	9	1.2%	To reduce dose of rosuvastatin in view of controlled lipid panel and elevated liver enzymes
				Adverse Drug Event	8	1.1%	Reduce dose of liraglutide in view of GI discomfort
				According to indication	9	1.2%	Loperamide should be given as initial dose of 4 mg followed by 2 mg as needed after bowel movements
				Drug-Drug Interaction	3	0.4%	To start escitalopram 5 mg during the last 2 days of tapering down quetiapine to prevent risk of QT prolongation or serotonin syndrome
				Drug-Disease Interaction	1	0.1%	Reduce dose of amantadine due to increased risk of lowering seizure threshold
	Optimum Administration	169	4.4%	Per guidelines/drug monographs/hospital protocols	56	33.1%	Separate calcium supplement from iron by 2 hours
				Treatment tapering	51	30.2%	To taper down nicotine patch strength to 7 mg after 2 weeks of 14 mg
				Optimise management	46	27.2%	To taper up dose of sertraline after one week in view of severe depressive symptoms
				Medication timing	16	9.5%	Reschedule amitriptyline to nighttime to prevent dizziness spells during the morning
	Optimum frequency	129	3.4%	Optimise management	64	49.6%	Reduce gabapentin frequency to once daily at bedtime
				Inpatient complication	25	19.4%	Depression, *n* = 28(36%); Spasticity/pain, *n* = 28(36%); Aphasia, *n* = 9(12%); Cognitive delay, *n* = 6(8%); Dizziness, *n* = 5(6%); Anxiety, *n* = 1(1%)
				Sequelae of disease	17	13.2%	Spasticity/pain, *n* = 11(73%); Cognitive delay, *n* = 2(13%); Anxiety, *n* = 1(7%); Dizziness, *n* = 1(7%)
				Per guidelines/hospital protocol	13	10.1%	Adjust frequency of oxybutynin to 5 mg BID due to immediate and short acting effect
				Uncontrolled condition	4	3.1%	Hypertension, *n* = 3(75%); Diabetes, *n* = 1(25%)
				Drug-Drug Interaction	2	1.6%	Switched prochlorperazine frequency to PRN in view of running regular order of metoclopramide
				Adverse Drug Event	2	1.6%	Switched pantoprazole to PRN in view of hyponatremia
				Liver function	1	0.8%	Switched paracetamol to PRN in view of elevated liver enzymes
				Weight	1	0.8%	Reduced frequency of prophylactic enoxaparin from BID to daily
	Inappropriate Duration	119	3.1%	According to indication	111	93.3%	To continue apixaban for total of 6 months post PE
				Drug-Drug Interaction	3	2.5%	Limit use of fluconazole for 5 days due to interaction with clopidogrel
				Optimise management	3	2.5%	Continue DAPT therapy for 3 weeks followed by aspirin lifelong
				Drug-Disease Interaction	2	1.7%	Limit use of NSAIDs to PRN for severe pain in patient with stable heart failure
Monitor (*n* = 482, 12.7%)	Appropriate Laboratory Recommended	393	10.3%	Labs for diagnosis	242	61.6%	Vitamin D level, *n* = 88 (22%); Glucose, *n* = 46 (12%); Electrolytes, *n* = 41 (10%); Anemia workup, *n* = 17 (4%); Thyroid function test, *n* = 16 (4%); CBC, *n* = 13 (3%); Lipid panel, *n* = 7 (2%); CMP, *n* = 5 (1%); Uric acid, *n* = 3 (1%); Inflammatory markers, *n* = 2 (1%); Platelet count, *n* = 2 (1%); Vitamin B level, *n* = 2 (1%
				Labs for kidney/liver function	125	31.8%	Kidney function, *n* = 71(57%); liver function, *n* = 54(43%)
				Labs for drug levels	15	3.8%	INR, *n* = 12(80%); Valproic acid level, *n* = 1(6.7%); carbamazepine levels, *n* = 1(6.7%);Vancomycin trough level, *n* = 1(6.7%)
				Labs for drug-drug interactions	11	2.8%	Monitor INR as steroid can increase serum concentration of warfarin
	Appropriate procedure recommended	29	0.8%	Consultation request	13	44.8%	Request cardiology consultation for patient with intracranial hemorrhage previously taking aspirin for CAD
				Culture	6	20.7%	Requesting urine culture due to elevated inflammatory markers
				CT scan	4	13.8%	To wait for CT results to inform on the discussion of continuing anticoagulation
				Specialised diet	2	6.9%	Recommend a low purine diet after gout attack
				ECG	1	3.4%	Monitor for QT prolongation
				Occult stool test	1	3.4%	
	Clinical signs and symptoms	27	0.7%	Drug-Drug Interaction	17	63.0%	Monitor for myopathy as colchicine may enhance statin effect
				Adverse Drug Event	10	37.0%	Monitor for symptoms of allergy after oral amoxicillin challenge
	Vital signs	24	0.6%				Monitor for blood pressure control after increasing dose of perindopril
	Discontinue inappropriate lab	9	0.2%	Not indicated	9	100.0%	Stop Blood sugar monitoring as patient is not diabetic
Medication therapy management (*n* = 290, 7.6%)	Periodic chart review	290	7.6%				
Drug Information (*n* = 188, 4.9%)	Drug Information Inquiry	188	4.9%	Optimum administration	53	28.2%	Tamsulosin to be given at nighttime to avoid postural hypotension
				Medication dosing	48	25.5%	To initiate nicotine therapy with 21 mg patches for 6 weeks as patient is heavy smoker (>10 cigarettes/ day)
				Medication selection	35	18.6%	Recommend cinnarizine for treatment of dizziness
				Drug-Drug Interaction	16	8.5%	Team requested to review possible interactions with memantine and current active treatments
				Allergy	12	6.4%	Possibility of using Aspirin in patient with allergy to celecoxib
				Adverse Drug Event	11	5.9%	Team requested to review current medications for a plausible cause of urinary retention
				Medication selection and dosing	9	4.8%	To initiate baclofen for hiccups
				Availability of product	2	1.1%	Requested to view whether a formulation of amlodipine/lisinopril is available within hospital's formulary
				Breastfeeding	1	0.5%	Compatibility of levetiracetam with breastfeeding
				Religious concern	1	0.5%	Request for information regarding Ramadan fasting and injectable drugs
Incomplete prescriptions (*n* = 6, 0.2%)	Missing weight	6	0.2%				
Patient education (*n* = 5, 0.1%)	Adherence	5	0.1%				

#### 3.2.1. Pharmacological strategy

The most frequently reported intervention under pharmacological strategy was recommending additional therapies to the treatment plan (*n* = 673, 17.7%). These recommendations were primarily aimed at addressing untreated conditions (*n* = 207, 30.7%), uncontrolled conditions (*n* = 154, 22.8%), sequalae of the primary disease (*n* = 113, 16.8%), inpatient complications (*n* = 100, 14.8%), adding prophylactic treatments (*n* = 87, 12.9%), or optimising patient care (*n* = 12, 1.8%).

Pharmacists also had a significant role in discontinuing medications (*n* = 476, 12.5%) for various reasons: the treatment was no longer needed (*n* = 211, 44.4%), the medication was not indicated (*n* = 124, 26.1%), there was a duplication of therapy (*n* = 85, 17.9%), adverse events had developed (*n* = 21, 4.4%), or there were interactions with diseases (*n* = 17, 3.6%), or other drugs (*n* = 13, 2.7%).

**Figure 1.**
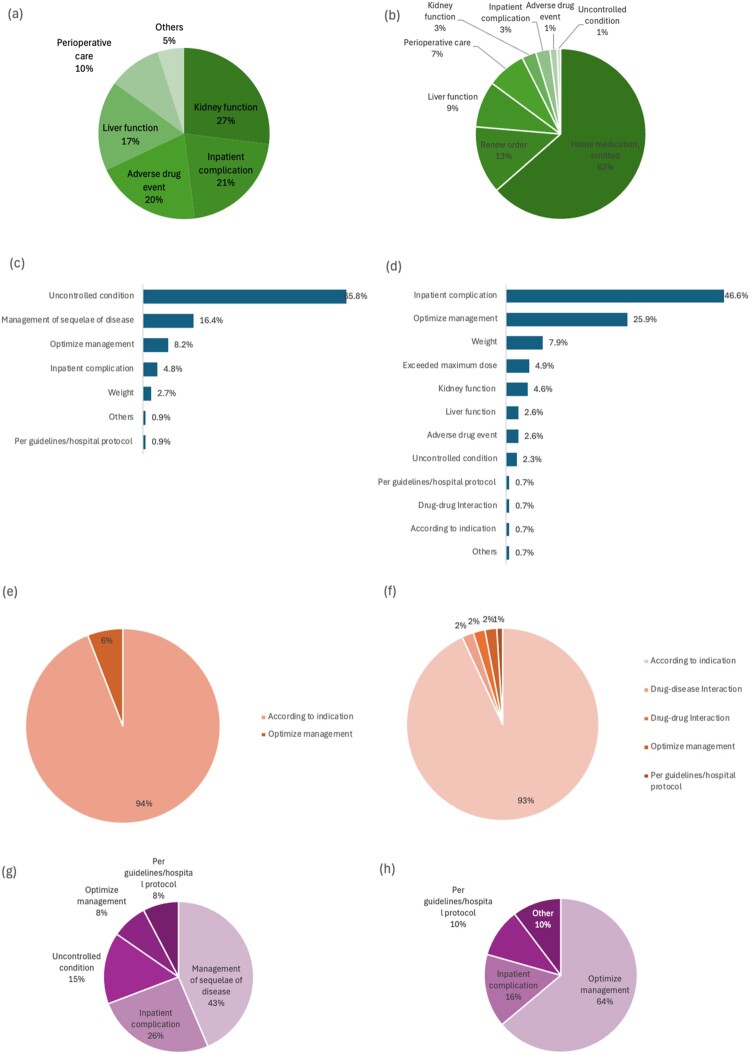
Type and reasons behind changes introduced by pharmacists’ interventions: (a) reasons to hold medications; (b) reasons to resume medications; (c) reasons to increase dose; (d) reasons to decrease dose; (c) reasons to increase duration; (d) reasons to decrease duration; (c) reasons to increase frequency; (d) reasons to decrease frequency.

Another role played by pharmacists was recommending holding (*n* = 96, 39.3%) or resuming (*n* = 148, 60.7%) medications as necessary. The main reason for resuming medications was omitted home medications on admission (*n* = 94, 63.5%) or renewal of discontinued orders (*n* = 19, 12.8%), while holding medications was judged by the deterioration of renal function (*n* = 27, 28.1%), development of inpatient complications (*n* = 21, 21.9%) adverse drug events (*n* = 20, 20.8%) or perioperative management (*n* = 10, 10.4%) (Figure 1(a,b)).

Recommending an alternative therapy was another subtype of the clinical pharmacists’ interventions (*n* = 230, 6%). The rationale behind the change was mainly to optimise the treatment plan (*n* = 82, 34.9%), availability of product (*n* = 69, 29.4%), kidney dysfunction (*n* = 20, 8.5%), development of adverse events (*n* = 14, 6%), drug-drug interactions (*n* = 14, 6%), or to tailor to culture results (*n* = 10, 4.3%).

Other interventions included adjustment of formulation selection (*n* = 47, 1.2%), which was mainly to account for enteral feeding tube placement (*n* = 14, 29.8%) or removal (*n* = 6, 12.8%).

#### 3.2.2. Quantity of drug

This type of intervention was the second most reported by pharmacists. It comprised intervening to optimise drug dose (*n* = 749, 19.7%), administration (*n* = 169, 4.4%), frequency (*n* = 129, 3.4%), and duration (*n* = 119, 3.1%) (Table 3). Addressing uncontrolled conditions (*n* = 298, 39.7%) and inpatient complications (*n* = 161, 21.5%) were the main reasons for intervening on medication dosing. Specifically, requesting a dose increase was addressed in 439 (59%) interventions, while dose reduction was requested in 305 (41%) interventions (Figure 1(c,d)).

Optimisation of medication administration was addressed in 4.4% of interventions (*n* = 169), where recommendations taken from guidelines/monographs/hospital protocols were provided in 56 (33.1%) interventions. Treatment tapering schedules (*n* = 51, 30.2%), medical management optimisation (*n* = 46, 27.2%), and medication administration timing (*n* = 16, 9.5%) were also addressed.

Frequency adjustments were also made, including increases (*n* = 39, 29%) and decreases (*n* = 96, 71%). Increasing frequency was needed primarily to address sequelae of primary disease (*n* = 17, 43.6%) and inpatient complications (*n* = 10, 25.6%) (Figure 1(g)); while frequency reduction was recommended mainly to optimise medical management (*n* = 62, 64.6%) or address inpatient complications (*n* = 15, 15.6%) (Figure 1(h)).

Lastly, pharmacists intervened to adjust an inappropriate treatment duration (*n* = 119, 3.1%) mainly to comply with durations needed for each medical indication (*n* = 109, 91.6%) (Table 3). To elaborate, pharmacists decreased the duration of regimens in 101 cases and increased the duration in 17 cases (Figure 1(e,f)).

#### 3.2.3. Monitor of laboratory values and diagnostics

Pharmacists addressed needed laboratory values and diagnostics in 10.3% (*n* = 393) of the interventions through suggestions and recommendations to physicians in the multidisciplinary team. Most interventions pertaining to monitoring revolved around laboratory follow up, especially for diagnosing or following up on certain disease states (*n* = 242, 61.6%), liver or kidney function tests (*n* = 125, 31.8%), drug levels (*n* = 15, 3.8%), or drug interactions (*n* = 11, 2.8%).

Other recommended procedures included requesting the assigned physician to contact specialty teams for consultations such as endocrine (*n* = 13, 44.8%). culture (*n* = 6, 20.7%), and imaging or electrocardiogram (ECG) (*n* = 5, 17.2%).

#### 3.2.4. Medication therapy management

Other interventions addressed by pharmacists in QRI is a periodic file review for long-term stable patients (*n* = 290, 7.6%), which includes a review of treatment plans and associated bundles of care (e.g. laboratory values) to ensure optimum care and absence of underlying or cryptic medical deterioration.

#### 3.2.5. Drug information inquiries

During the study period, clinical pharmacists responded to 188 (4.9%) drug information queries. The most common causes for inquiries were related to the optimum administration (*n* = 53, 28.2%), optimum dosing (*n* = 48, 25.5%), selection of medications (*n* = 35, 18.6%), presence of drug-drug interactions (*n* = 16, 8.5%), approach to allergic patients (*n* = 12, 6.4%), or handling of adverse events (*n* = 11, 5.9%).

### 3.3. Drug categories associated with intervention

**Figure 2.**
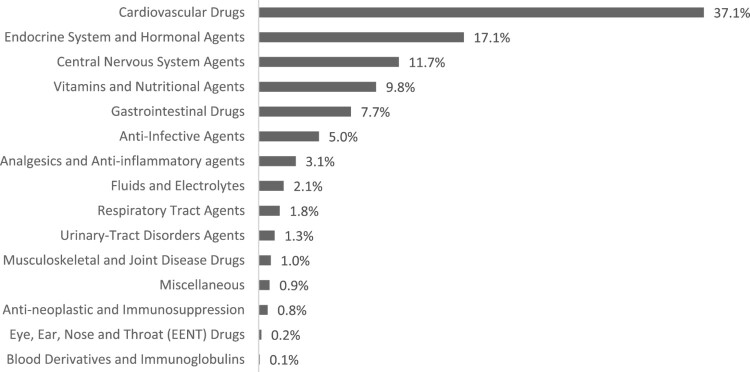
Categories of drugs addressed by pharmacists’ interventions.

Drug categories targeted in the interventions are depicted in Figure 2 (for detailed description, see Supplemental Table S1). As multiple interventions targeted non-drug related issues (i.e. laboratory values or procedures) it was expected that the total number of implicated drugs would not match the total number of interventions. Most interventions were on cardiovascular agents (*n* = 1137, 37.1%), namely hypertensives (*n* = 683, 60.1%) and anticoagulants (*n* = 221, 10.1%). Endocrine and hormonal agents (*n* = 524, 17/1%), primarily anti-diabetics (*n* = 483 (92.2%), were the second most addressed group of drugs.

A considerable focus on central nervous system agents has been noted (*n* = 358, 11.7%), mainly by addressing issues relating to selective serotonin and serotonin/norepinephrine reuptake inhibitors (SSRI/SNRI) (*n* = 112, 31.3%) and anti-epileptics (*n* = 84, 23.5%). Addressing neurological sequalae, specifically medications targeting aphasia (*n* = 50, 14%) or neurostimulation (*n* = 51, 14.2%) were also noted.

Pharmacists’ interventions also encompassed other drug categories, including adding needed vitamins (*n* = 300, 9.8%), gastrointestinal drugs (*n* = 236, 7.7%), and analgesics (*n* = 95, 3.1%). Additionally, anti-infective drugs (*n* = 154, 5%), particularly antibiotics (*n* = 138, 89.6%), were addressed.

### 3.4. Severity of interventions

**Figure 3.**
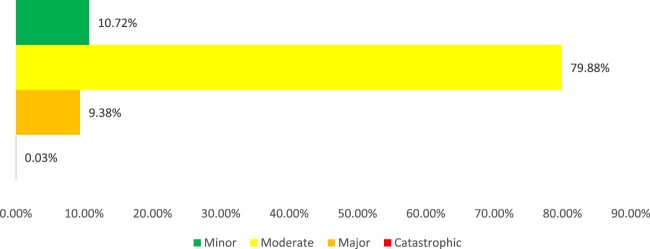
Severity of pharmacists’ interventions.

The severity of interventions is presented in Figure 3. Explanatory examples of different levels of severity are reported in Supplemental Table S2.

A total of 3041 of the interventions (79.88%) were classified as moderate in nature (related to cardiovascular agents (*n* = 879, 28.9%), endocrinology drugs (*n* = 432, 14.2%), centrally-acting agents (*n* = 414, 13.6%), and laboratory tests (*n* = 373, 12.3%)). Second most reported significance level was minor (*n* = 408, 10.72%); mostly on vitamins and nutritional agents (*n* = 298, 73%) and gastrointestinal drugs (*n* = 26, 6.4%). Major interventions constituted 9.38% (*n* = 357), mainly targeting cardiovascular drugs (*n* = 216, 60.5%) and anti-infective agents (*n* = 84, 23.5%). Only one intervention was described as catastrophic, which involved identification of premature discontinuation of rivaroxaban indicated for a provoked extensive DVT after the patient was transferred back and forth for a cranioplasty procedure. No statistical difference was noted between elderly and adults in terms of interventions significance levels (*p* = 0.09).

### 3.5. Prescribers’ response

During the study period, the prescribers accepted 95.9% (*n* = 3651) of pharmacists’ interventions while 4.1% (*n* = 156) were rejected (see Supplemental Table S2). The most frequently rejected type of intervention was dose optimisation (*n* = 41, 26.3%), followed by requests for adding a new therapy (*n* = 35, 22.4%), discontinuing a therapy (*n* = 28, 17.9%), and recommending an alternative therapy (*n* = 24, 15.4%) (3).

## 4. Discussion

### 4.1. Key findings

A total of 3807 clinical pharmacists’ interventions performed in 2023 were collected and analysed in this study. Findings indicate that the majority of interventions were addressing pharmacological strategy (*n* = 1670, 43.9%), followed by drug quantity (*n* = 1166, 30.7%). Subtypes of interventions most frequently documented were optimising dose (*n* = 749; 19.7%), recommending additional drug therapy (*n* = 673; 17.7%), discontinuation of medication (*n* = 476; 12.5%) and recommending appropriate laboratory tests (*n* = 393; 10.3%). The main reasons for the interventions included managing uncontrolled conditions (e.g. diabetes), optimisation of medical management (e.g. optimising dosing of warfarin), addressing inpatient complications (e.g. electrolyte imbalance), recommending diagnostic laboratory tests (e.g. electrolytes), and managing sequelae of primary disease (e.g. spasticity, depression). Cardiovascular agents were involved in 37.1% of the interventions, followed by endocrine medications (17.1%) and centrally-acting agents (11.7%). Most interventions were of moderate severity (79.8%). Our statistical analyses showed that age had a positive linear correlation with the number of interventions per patient (*p* < 0.001); however, no difference was seen between adult and elderly patients in term of the severity of interventions (*p* = 0.09).

This study highlighted pharmacological strategy and drug quantity management as the two most frequent areas of pharmacist’s intervention in a rehabilitation setting. Although one randomised controlled trial demonstrated that pharmacist participation in a hospital multidisciplinary rehabilitation team significantly reduced the number of DRPs, with benefits persisting after discharge, this study did not analyse the nature and severity of interventions performed (Willoch et al., 2012). This highlights the urgent need to characterise and quantify clinical pharmacy contributions to rehabilitation programmes to better understand the current roles pharmacists play in mitigating errors and to identify potential areas of improvement. A study by W. Brown (1994) similarly described the integration of pharmacists into multidisciplinary rehabilitation teams, reporting similar intervention types (Brown, 1994). Of 167 patient encounters, the primary reasons for pharmacist-initiated modifications were discontinuing unnecessary medication (33.5%), correcting improper dosage (16.2%), and addressing untreated conditions (11.3%). A published abstract from neurological rehabilitation units further supports this, with discontinuation (33%) and dose adjustments (22%) being the most commonly suggested pharmacist interventions (Hubault et al., 2015). Additionally, two studies focused on post-intensive care rehabilitation clinics observed a similar trend, even though they employed different classification systems for interventions (MacTavish et al., 2019; Stollings et al., 2023).

The findings of this study emphasise the valuable role pharmacists can play in enhancing the rehabilitation process by assessing and managing untreated or uncontrolled conditions such as anemia, diabetes, and hypertension. These conditions are common among patients undergoing rehabilitation and can severely hinder recovery if not addressed properly. For example, hypertension and diabetes can impair physical stamina, prolong recovery times, and lead to declines in both cognitive and physical function, all of which can negatively impact rehabilitation outcomes (Kuo et al., 2005). Similarly, several studies have established the crucial role of nutritional support – such as improving hemoglobin levels and supplementing with vitamin D and B – in accelerating recovery, enhancing patients’ functional capacity, and reducing hospital stay durations (Clements et al., 2020; Mizuno et al., 2022; Smith-Ryan et al., 2020; Wojzischke et al., 2020; Yoshimura et al., 2021). The positive impact of pharmacist on nutritional support has been demonstrated in a previous study that evaluated the appropriateness of enteral feeding through a permanent ostomy in non-ambulatory patients with severe developmental disabilities (Brown et al., 1997).

Additionally, clinical pharmacists actively contribute to optimising the treatment of complications that arise from the primary condition, such as spasticity, pain, depression, aphasia, and cognitive impairment. In patients recovering from strokes or traumatic brain injuries, for example, pharmacists play a pivotal role in adjusting medications to manage spasticity or neuropathic pain, which can otherwise impede physical therapy progress. Addressing psychological complications like depression or anxiety is equally crucial for keeping patients engaged in their rehabilitation programmes. This highlights the potential positive role of a clinical pharmacist not only in enhancing clinical outcomes but also in improving the overall rehabilitation outcomes. This includes improved primary rehabilitation goals such as activities of daily living (ADL) and cognitive activities of daily living (CADL) and subsequently improving patient’s quality of life upon discharge (Kose & Wakabayashi, 2020; Lee et al., 2020; Yoshimura et al., 2022).

Several renowned pharmacy societies highlight the tenet of medication therapy management, which involves assessing, evaluating, and monitoring patients’ response to therapy (Hospital Pharmacy in Canada Editorial Board, 2013; Myers, 2004). This practice is particularly crucial for long-term hospitalised patients, such as those in rehabilitation units where pharmacotherapy management must address both the primary condition that necessitated hospitalisation and any complications that arise during the hospital stay (Yoshimura et al., 2022). This aligns with one of the primary tasks for clinical pharmacists at QRI: the periodic file review. During this process, pharmacists conduct a comprehensive reevaluation of patients’ electronic charts, reviewing physician notes, physiotherapy notes, active medications, and laboratory values. Their findings are then documented and discussed with the primary care provider to modify treatment plans as necessary. This approach ensures that pharmacists are proactively involved in optimising pharmacotherapeutic treatment plans, contributing to more effective patient care.

The literature consistently highlights the importance of pharmacotherapy management in addressing potentially inappropriate medications and polypharmacy, particularly in long-term hospitalised patients, who are often elderly (Clarke & Witham, 2017; Lee et al., 2020). Notably, this study found a small number of elderly patients admitted to QRI, which may be attributed to the country's ethnic distribution (National Planning Council State of Qatar, 2024), varying risk of stroke (Turana et al., 2021), and the involvement of motor vehicle accidents – two major indications for rehabilitation in Qatar. Despite the median age of patients in this study, deprescribing was a central focus of pharmacists’ interventions, underscoring the ongoing need for regular assessment of active treatments to manage and prevent unnecessary polypharmacy, especially in long-term rehabilitation settings (Kose & Wakabayashi, 2020). Deprescribing is critical not only for minimising adverse drug reactions but also for improving patient outcomes by reducing the burden of unnecessary medications, improving adherence and enhancing QoL (Linsky et al., 2023).

Consistent with studies conducted in various settings, dose adjustment was among the most commonly reported interventions in our study (Al Rahbi et al., 2014; Alzahrani et al., 2021; Naseralallah et al., 2020, 2024). However, it is important to note that, in most other settings, pharmacists typically recommended decreasing medication doses due to factors such as altered kidney function, age, or weight. In contrast, our study revealed that the majority of recommendations – approximately 60% – were to increase doses to optimise the management of uncontrolled chronic conditions or complications. For instance, increasing doses may have been necessary to achieve therapeutic targets for conditions like hypertension or diabetes, where inadequate control could potentially lead to significant morbidity and complicate rehabilitation efforts. This further highlights the vital role pharmacists play in improving both clinical and rehabilitation outcomes. Moreover, it is noteworthy that pharmacists still recommended dose reductions in about 40% of cases, demonstrating their active involvement in maintaining safe treatment protocols and reinforces their commitment to patient safety. Such balanced interventions reflect pharmacists’ critical role in navigating the complexities of pharmacotherapy, particularly in patients with multiple comorbidities who may be at risk of polypharmacy. Clinical pharmacists’ ability to make nuanced recommendations based on individual patient profiles ensures that treatment plans are not only effective but also safe, ultimately enhancing the quality of care provided to patients in rehabilitation.

### 4.2. Strengths and limitations

This research was conducted at the only hospital dedicated to rehabilitation in Qatar. It is considered the first study in literature to provide a comprehensive evaluation of pharmacists’ interventions using a systematic and meticulous approach to analyse and categorise interventions from a reporting database specific to this population. Despite this rigorous methodology, inherent subjectivity may arise in the classification process, as there is often insufficient detail in the free-text descriptions. Moreover, since it is the single hospital targeting neurological and musculoskeletal rehabilitation in Qatar, generalisability of the results may be limited; however, this is the sole hospital in Qatar with this setting; given the paucity of literature describing the clinical pharmacists’ interventions in this setting, it was deemed significant contribute to the literature through QRI’s practice. Additionally, documenting interventions is a mandatory task for clinical pharmacists under HMC policy, as it is a key criterion in their annual evaluation. This requirement could potentially lead to overreporting of clinically irrelevant interventions. Finally, this study did not assess the impact of these interventions on relevant clinical outcomes.

### 4.3. Future directions

This study strongly supports the role of clinical pharmacists in patients’ rehabilitation journeys, highlighting the significant opportunities they have to improve both clinical and rehabilitation outcomes. Therefore, it is essential to encourage pharmacists to obtain relevant specialised training that addresses the unique needs of this setting. This training should include tailored clinical skills, such as deprescribing and identifying opportunities to enhance patients’ health status, as well as a solid foundation in general pharmacology with a focus on psychopharmacology. Furthermore, stakeholders and policymakers should be proactive in raising funding and facilitating pharmacy career development, ensuring that more specialty opportunities are available to pharmacists in this field.

The role of pharmacists in rehabilitation settings is rarely covered in the literature; thus, future observational studies are essential to better understand the impact of clinical pharmacists in this context. Additionally, future research should address potential methodological biases present in this study by incorporating qualitative approaches, such as focus groups or semi-structured interviews, with the multidisciplinary rehabilitation team to explore the potential role and impact of clinical pharmacists more deeply. Finally, it is crucial to assess the impact of pharmacists’ interventions on clinically significant outcomes, including length of stay, polypharmacy, QoL, and improvements in ADL. Conducting cost-effectiveness analyses will also be important for helping policymakers make informed decisions about integrating pharmacists into rehabilitation settings.

## 5. Conclusion

This study highlights the diverse roles of clinical pharmacists in the rehabilitation field and suggests that they can significantly improve both clinical and rehabilitation outcomes. The unique complexity of rehabilitation patients presents a challenging environment for clinical pharmacists, necessitating adherence to fundamental practice principles while also customising approaches to meet individual patient needs. Further research is essential to evaluate the impact of these interventions on clinically significant outcomes.

## Supplementary Material

supplementary.docx
