# Supplementary material for: Analysis of clinical pharmacists’ interventions in a rehabilitation setting
Source: J Pharm Policy Pract. 2025 Apr 25;18(1):2450593. doi: 10.1080/20523211.2025.2450593 (PMC12035954; doi:10.1080/20523211.2025.2450593)
Supplement: supplementary.docx [file JPPP_A_2450593_SM3773.docx]

| Table S1. Drug categories addressed in evaluated interventions | | | | | |
| --- | --- | --- | --- | --- | --- |
| **Cardiovascular Drugs** | **1137** | **37.1%** | **Anti-Infective Agents** | **154** | **5.0%** |
| *HTN* | *683* | 60.1% | *Antibiotics* | *138* | 89.6% |
| ACE-i/ARB | 242 | 21.3% | Penicillin | 30 | 19.5% |
| CCB | 147 | 12.9% | Nitrofurantoin | 22 | 14.3% |
| BB | 146 | 12.8% | Cephalosporin | 19 | 12.3% |
| Hydralazine | 62 | 5.5% | Others | 15 | 9.7% |
| Diuretics | 48 | 4.2% | TB medications | 11 | 7.1% |
| others | 38 | 3.3% | Carbapenems | 9 | 5.8% |
| *Anticoagulant* | *221* | 10.1% | Metronidazole | 9 | 5.8% |
| injectable | 178 | 19.4% | Fluoroquinolones | 7 | 4.5% |
| DOAC | 43 | 15.7% | Glycopeptides | 7 | 4.5% |
| *Antiplatelet* | 115 | 3.8% | Trimethoprim-sulfamethoxazole | 6 | 3.9% |
| *Cholesterol* | *72* | 6.3% | Macrolide | 3 | 1.9% |
| *Others* | *46* | 4.0% | *Antifungals* | *12* | 7.8% |
| **Endocrine System and Hormonal Agents** | **524** | **17.1%** | *Others* | *4* | 2.6% |
| Anti-Diabetics | 483 | 92.2% | **Analgesics and Anti-inflammatory agents** | **95** | **3.1%** |
| Steroids | 25 | 4.8% | NSAIDs | 54 | 56.8% |
| Hormonal agents | 6 | 1.1% | Paracetamol | 26 | 27.4% |
| Osteoporosis | 6 | 1.1% | Opioids | 15 | 15.8% |
| Miscellaneous | 4 | 0.8% | **Fluids and Electrolytes** | **64** | **2.1%** |
| **Central Nervous System Agents** | **358** | **11.7%** | Sodium supplementation | 23 | 35.9% |
| SSRI/SNRI | 112 | 31.3% | Magnesium supplementation | 18 | 28.1% |
| Anti-seizure | 84 | 23.5% | IV fluids | 6 | 9.4% |
| Neurostimulation | 51 | 14.2% | Potassium supplementation | 6 | 9.4% |
| Aphasia | 50 | 14.0% | Calcium supplementation | 5 | 7.8% |
| Miscellaneous | 35 | 9.8% | Bicarbonate supplementation | 4 | 6.3% |
| Anxiolytics | 18 | 5.0% | Calcium polystyrine sulfonate | 2 | 3.1% |
| Antipsychotics | 8 | 2.2% | **Respiratory Tract Agents** | **54** | **1.8%** |
| **Vitamins and Nutritional Agents** | **300** | **9.8%** | **Urinary-Tract Disorders Agents** | **41** | **1.3%** |
| **Gastrointestinal Drugs** | **236** | **7.7%** | **Musculoskeletal and Joint Disease Drugs** | **39** | **1.0%** |
| PPI | 110 | 46.6% | Baclofen | 30 | 76.9% |
| Anti-nausea/vomiting | 49 | 20.8% | Tizanidine | 5 | 12.8% |
| Laxatives | 47 | 19.9% | Botulinum toxin A | 4 | 10.3% |
| Miscellaneous | 21 | 8.9% | **Anti-neoplastic and Immunosuppression** | **23** | **0.8%** |
| Symptomatic agents | 9 | 3.8% | Biologics | 11 | 47.8% |
| **Eye, Ear, Nose and Throat (EENT) Drugs** | **7** | **0.2%** | IVIG | 8 | 34.8% |
| **Blood Derivatives and Immunoglobulins** | **3** | **0.1%** | Others | 5 | 21.7% |
|  |  |  | **Miscellaneous** | **28** | **0.9%** |

| **Table S2. Type of interventions rejected by prescribers (n=156)** |  |  |
| --- | --- | --- |
| **Pharmacological strategy** | **103** | 66.0% |
| Additional Therapy Required | 35 | 22.4% |
| Alternative Therapy | 24 | 15.4% |
| Discontinue medication | 28 | 17.9% |
| Hold/Resume | 14 | 9.0% |
| Formulation Selection | 2 | 1.3% |
| **Quantity of drug** | **48** | 30.8% |
| Optimum Dose | 41 | 26.3% |
| Optimum Administration | 3 | 1.9% |
| Optimum frequency | 3 | 1.9% |
| Inappropriate Duration | 1 | 0.6% |
| **Monitor** | **5** | 3.2% |
| Appropriate Laboratory Recommended | 3 | 1.9% |
| Appropriate procedure recommended | 2 | 1.3% |
